# The TABLET trial: limitations and implications

**DOI:** 10.1186/s12916-019-1365-y

**Published:** 2019-07-10

**Authors:** Chrysoula Dosiou, Alex Stagnaro-Green

**Affiliations:** 1 Division of Endocrinology, Stanford University School of Medicine, 300 Pasteur Drive, Room S025, Stanford, CA 94305 USA; 2 Internal Medicine, Obstetrics and Gynecology, and Medical Education, University of Illinois College of Medicine at Rockford, 1601 Parkview Avenue, Rockford, IL 61107 USA

**Keywords:** Miscarriage, Levothyroxine treatment, Thyroid autoimmunity, TABLET trial

## Background

The link between thyroid antibodies and spontaneous pregnancy loss was published in a seminal paper in 1990. In this study of a prospective cohort of unselected women, the rate of miscarriage was twice as high in euthyroid thyroid antibody-positive women than in euthyroid thyroid antibody-negative women [1]. A 2011 meta-analysis of cohort studies replicating this finding reported the odds of pregnancy loss to be over three times higher in euthyroid thyroid antibody-positive women [2]. A critical question remains: what is the impact of intervention with levothyroxine on the rate of miscarriage in euthyroid thyroid antibody-positive women? In 2006, the first randomized controlled trial (RCT) reported a significant decrease in the miscarriage rate (from 13.8 to 3.5%, *P* < 0.05) in unselected euthyroid women with thyroid autoimmunity treated with levothyroxine compared with the untreated group [3].

## The thyroid antibodies and levothyroxine (TABLET) trial

The TABLET trial is a large, multicenter, double-blind RCT of levothyroxine treatment versus placebo, in euthyroid thyroid peroxidase antibody (TPO Ab)-positive women, carried out in 49 centers in the UK. The study enrolled 952 women with a history of miscarriage or infertility, aged 16–40, and trying to conceive within the next year. Subjects were treated pre-pregnancy with a fixed dose of levothyroxine (50 μg) and continued that treatment throughout gestation. Primary outcome was live births after 34 weeks of gestation; secondary outcomes included miscarriage, preterm delivery, and neonatal outcomes. The study showed no significant difference between the two groups in any of the outcomes [4].

This study has several important strengths. First, it had a large sample size and was adequately powered to detect a difference in the primary outcome. Second, treatment with levothyroxine was started before conception. Third, high rates of follow-up (98.7%) were achieved for the primary outcome. Finally, subgroup analyses were performed with respect to important variables.

However, the study has some significant limitations. First, the dose of levothyroxine was fixed at 50 μg and was not adjusted during pregnancy, as per the American Thyroid Association guidelines [5]. Second, the TPO Ab test was not standardized, but run on 22 different analyzers, with borderline or equivocal results considered positive. This could have resulted in some women without thyroid autoimmunity being misclassified in the TPO Ab-positive category. Third, anti-thyroglobulin antibodies were not measured, which could mean that some women with thyroid autoimmunity were missed. Fourth, thyroid stimulating hormone (TSH) levels were quite similar in the levothyroxine-treated group versus the placebo group (median TSH 1.73 versus 1.94, respectively, at 9 months after randomization; 1.31 versus 1.60 at 16–18 weeks of gestation; and 1.30 versus 1.50 at 28 weeks of gestation). Fifth, the population of women studied was not uniform: it included women with a history of either infertility or miscarriage, with 20% of women overall having a history of recurrent pregnancy loss. Sixth, women were included whether they conceived spontaneously or through assisted reproduction. Finally, rates of miscarriage were unusually high in both groups compared with prior studies [3, 6], limiting the generalizability of the results.

The study results are in conflict with a previous RCT in euthyroid TPO Ab-positive women by Negro and colleagues. An important difference between the two studies is the population of women included. The Negro study consisted exclusively of unselected women, whereas only women with a history of miscarriage or infertility were studied in the TABLET trial. Furthermore, in the Negro study, women conceived spontaneously, whereas in the TABLET study about half of the women underwent treatment for infertility. A recent meta-analysis found that the benefit of levothyroxine in reducing miscarriages in euthyroid women with thyroid autoimmunity might only be present in women who conceive naturally, and not in those using assisted reproduction [7]. The dosing of levothyroxine was also more aggressive in the Negro study, based on TSH level and TPO Ab titer initially versus a fixed dose in the TABLET trial. This resulted in TSH levels that were significantly lower in the intervention group (1.1 versus 2.3 mIU/L) and similar to the TPO Ab-negative control group (1.2 mIU/L) at 20 weeks of gestation [3]. Finally, the miscarriage rates were lower in the Negro study (approximately 14% versus 30% in the TABLET trial), but more representative of the percentage found in the general population [6].

## Implications

The TABLET study results, along with those of the recently published Pregnancy Outcomes Study in Euthyroid Women With Thyroid Autoimmunity after Levothyroxine (POSTAL) study [8], an RCT that showed no effect of levothyroxine on pregnancy outcomes in euthyroid TPO Ab-positive women undergoing in vitro fertilization treatment, raise intriguing questions on thyroid antibody positivity and pregnancy loss. The lack of impact of levothyroxine in the TABLET trial supports the concept that the primary pathogenesis of pregnancy loss in euthyroid women with thyroid autoimmunity is a hostile immune environment [1], not a relative thyroid hormone deficiency. The recent discovery of TPO expression on mature granulosa cells [9] lends support to this hypothesis. Alternatively, the pathogenesis of miscarriage may differ in distinct subsets of women; for example, women with recurrent pregnancy loss or specific reasons for infertility. It is also possible that women conceiving through assisted reproduction techniques respond differently than women with spontaneous pregnancies [7]. It is therefore feasible that only subgroups of euthyroid women with thyroid autoimmunity would benefit from levothyroxine, such as unselected women, women with certain causes of infertility, spontaneous pregnancies, higher TSH levels or higher TPO Ab titers, or women with impaired response to human chorionic gonadotropin [10].

## Conclusions

The TABLET trial, which shows no effect of levothyroxine on live birth rates in euthyroid TPO Ab-positive women, raises important questions regarding the pathophysiology of miscarriage in these patients, but has significant limitations. Future studies in this field must be carefully designed to ensure a correctly classified and well-defined study population, and provide levothyroxine so that it results in meaningful differences in thyroid function between studied groups. Meanwhile, we hope the TABLET study results will fuel further investigation into the basic pathophysiology of miscarriage in euthyroid women with thyroid autoimmunity that will lead to smarter, more targeted, and more effective approaches to treatment.

## Data Availability

Not applicable.
